# *PPIP5K2* and *PCSK1* are Candidate Genetic Contributors to Familial Keratoconus

**DOI:** 10.1038/s41598-019-55866-5

**Published:** 2019-12-18

**Authors:** Mariam Lofty Khaled, Yelena Bykhovskaya, Chunfang Gu, Alice Liu, Michelle D. Drewry, Zhong Chen, Barbara A. Mysona, Emily Parker, Ryan P. McNabb, Hongfang Yu, Xiaowen Lu, Jing Wang, Xiaohui Li, Abdulrahman Al-Muammar, Jerome I. Rotter, Louise F. Porter, Amy Estes, Mitchell A. Watsky, Sylvia B. Smith, Hongyan Xu, Khaled K. Abu-Amero, Anthony Kuo, Stephen B. Shears, Yaron S. Rabinowitz, Yutao Liu

**Affiliations:** 1 Department of Cellular Biology and Anatomy, Augusta University, Augusta, GA USA; 2 Department of Biochemistry, Faculty of Pharmacy, Cairo University, Cairo, Egypt; 3 Department of Surgery and Regenerative Medicine Institute, Cedars-Sinai Medical Center, Los Angeles, CA USA; 4 Inositol Signaling Group, Signal Transduction Laboratory, National Institute of Environmental Health Sciences, Research Triangle Park, NC USA; 5 Department of Ophthalmology, Duke University Medical Center, Durham, NC USA; 6 James and Jean Culver Vision Discovery Institute, Augusta University, Augusta, GA USA; 7 Institute for Translational Genomics and Population Sciences, Los Angeles Biomedical Research Institute and Department of Pediatrics and Medicine at Harbor-UCLA, Torrance, CA USA; 8 Department of Ophthalmology, Glaucoma Research Chair, King Saud University, Riyadh, Saudi Arabia; 9 Department of Eye and Vision Science, University of Liverpool, and St Paul’s Eye Unit, Royal Liverpool Hospital, Liverpool, UK; 10 Department of Ophthalmology, Augusta University, Augusta, GA USA; 11 Department of Population Health Science, Augusta University, Augusta, GA USA; 12 Center for Biotechnology and Genomic Medicine, Augusta University, Augusta, GA USA

**Keywords:** Corneal diseases, Vision disorders, Experimental models of disease, Genetics research

## Abstract

Keratoconus (KC) is the most common corneal ectatic disorder affecting >300,000 people in the US. KC normally has its onset in adolescence, progressively worsening through the third to fourth decades of life. KC patients report significant impaired vision-related quality of life. Genetic factors play an important role in KC pathogenesis. To identify novel genes in familial KC patients, we performed whole exome and genome sequencing in a four-generation family. We identified potential variants in the *PPIP5K2* and *PCSK1* genes. Using *in vitro* cellular model and *in vivo* gene-trap mouse model, we found critical evidence to support the role of PPIP5K2 in normal corneal function and KC pathogenesis. The gene-trap mouse showed irregular corneal surfaces and pathological corneal thinning resembling KC. For the first time, we have integrated corneal tomography and pachymetry mapping into characterization of mouse corneal phenotypes which could be widely implemented in basic and translational research for KC diagnosis and therapy in the future.

## Introduction

Keratoconus (KC, OMIM 14830) is a bilateral, asymmetric corneal degeneration characterized by localized thinning and protrusion of the thinned cornea, which can lead to high myopia, irregular astigmatism, and cornea scarring^1,2^. It has been reported that genetic, environmental, biomechanical, hormonal, enzymatic, and possibly inflammatory factors contribute to the pathogenesis of KC^2–4^. Although the majority of KC cases are sporadic, the genetic contribution in KC pathogenesis has been validated by various studies^5^. Familial inheritance was reported in 6–23.5% of KC patients, and first-degree relatives are at 15–67 times higher risk than the general population^6^. Familial KC is mostly inherited through autosomal dominant and occasionally recessive patterns^6,7^. More concordance of KC severity has been reported in monozygotic (MZ) twins than dizygotic twins (DZ)^5,8,9^, and consanguineous marriage (first-cousin marriage) is a risk factor for KC^10,11^. KC was also reported to be associated with chromosomal abnormality disorders such as Down syndrome (trisomy 21) and 22q11.2 deletion syndrome^12–15^. Furthermore, KC is associated with multiple genetically inherited disorders including Leber congenital amaurosis^16,17^, cataract^18^, retinal cone dystrophy^19^, Ehlers–Danlos syndrome, and osteogenesis imperfecta^20,21^.

Genome-wide linkage analyses have identified a number of genomic loci linked with KC. These include 1p36.23–36.21, 2p24, 2q13, 3p14-q13, 5q14.3-q21.1, 5q21.2, 5q32-q33, 8q13.1-q21.11, 9q34, 13q32, 14q11.2, 14q24.3, 15q15.1, 15q22.33–24.2, 16q22.3-q23.1, and 20p13-p12.2, 20q12^22^. However, the exact disease-causing mutations remain unknown for most patients^23^. Intriguingly, the linkage locus 5q21.2 has been replicated in two independent studies^24,25^. Follow-up studies have identified many potential mutations segregating with KC including *MIR184 c.57* *C* > *T*^18,26–32^. The mutation *MIR184 c.57* *C* > *T* was identified in the linkage region 15q22.32–24.2 in a Northern-Irish family who suffered from KC and congenital cataract through targeted sequencing-capture^18,33,34^. *MIR184 c.57* *C* > *T* mutation has been functionally validated by an *in vitro* model^18^. However, *in vitro* and/or *in vivo* models are necessary to elucidate the potential molecular impact of identified mutations in KC pathogenesis.

Targeted sequencing has been successful in elucidating the causative genetic mutation(s) in numerous inherited diseases such as neurofibromatosis type 1, Marfan syndrome, dilated cardiomyopathy, congenital disorders of glycosylation, and KC^35,36^. However, targeted sequencing relies on the prior identification of a significant linkage locus. The advent of whole exome sequencing (WES) or whole genome sequencing (WGS) technology offers a significant advantage by interrogating all the coding or genome sequence in the absence of a linkage locus^35^. WES is often used to identify variants in the protein-coding regions (about 1% of human genome), while WGS explores variants in the whole genome as well as structural changes such as copy number variants. We have successfully used WES and WGS to identify pathogenic variant(s) in a four-generation KC family with a reported linkage locus chr5q14.3–21.1 with autosomal dominant inheritance^24,37^.

## Results

### Sequence variants identified in the KC family linked to chr5q14.3–21.1

**Figure 1:**
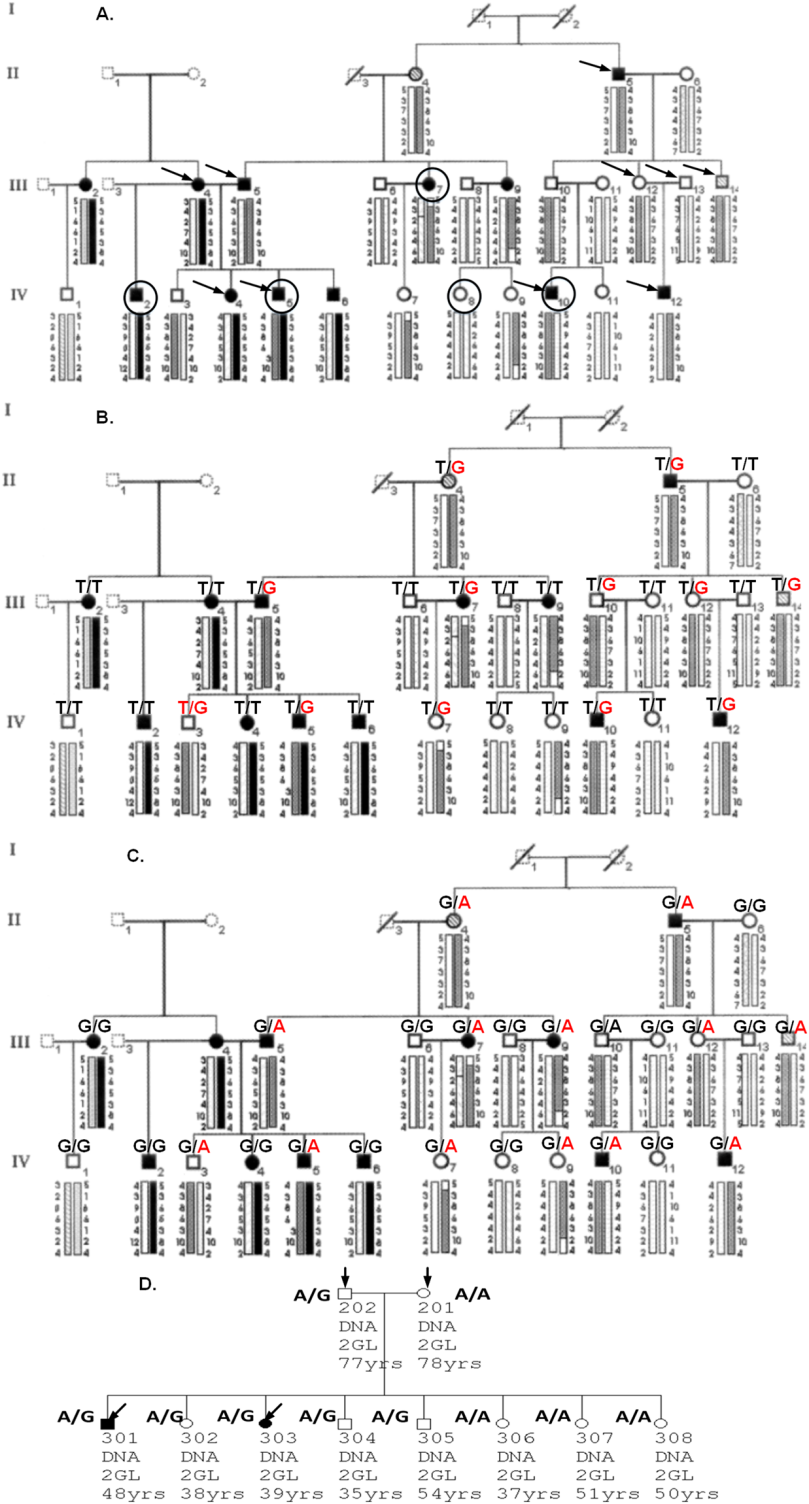
The pedigree structure and the genotypes of identified pathogenic variants in keratoconus-affected multiplex families with autosomal dominant inheritance using whole exome and whole genome sequencing. (**A**) The pedigree structure of the four-generation family with keratoconus. Generations are indicated by the Roman numbers I, II, III, and IV on the left side. Individuals in each generation are labeled with Arabic numbers 1 to 14. Linkage haplotypes are labeled under each individual. The gray haplotype is linked with keratoconus. Individuals with dashed lines were not enrolled in the genetic study, while those with the solid lines were consented and enrolled. Subjects with a black arrow were selected for whole exome sequencing while those with a black circle were selected for whole genome sequencing. (**B**) The alleles are indicated as major allele/minor allele. *PPIP5K2* variant rs35471301, p.Ser419Ala, S419A (T/G). Two individuals (III9 and IV9) carrying the linkage haplotype indicated by a grey bar are homozygotes for the major allele T. (**C**) *PCSK1* variant rs373951075 (G/A). All individuals with KC carrying the linkage haplotype indicated by grey bar are heterozygous for the minor allele A. (**D**) The pedigree structure of a second unrelated multiplex family and the genotypes of a pathogenic variant in the *PPIP5K2* gene. Subjects with a black arrow were selected for whole exome sequencing and bioinformatics analysis. Additional individuals were analyzed by PCR-based Sanger sequencing. Allele G is the minor allele.

In the four-generation multiplex family previously linked to chr5q14.3–21.1^24,37^, we selected ten individuals (KC patients: II5, III4, III5, IV4, IV5, IV10, and IV12; controls: III12, III13, and III14) for WES sequencing (Fig. 1A, with selected individuals indicated by a black arrow). After no relevant candidate variants were identified in a narrow 95–100 Mb region identified by both linkage and association analysis^37^, we extended the targeted region to cover the original linkage peak^24^ and identified a non-synonymous variant rs35671301 (chr5:103,154,707, c. 1255 T > G, p.Ser419Ala, S419A) located in the *PPIP5K2* (Diphosphoinositol Pentakisphosphate Kinase 2) gene. This is potentially a significant observation, as PPIP5K2 is a bi-functional kinase/phosphatase that controls the cell-signaling activities of the inositol pyrophosphates, InsP7 (diphosphoinositol pentakisphosphate) and InsP8 (bis-diphosphoinositol tetrakisphosphate)^38–40^. This variant co-segregates with KC in all 10 individuals sequenced with WES in this family. This variant was categorized as a rare change with a global MAF (minor allele frequency) of 0.0069 in the GnomAD database (genome aggregation database, gnomad.broadinstitute.org). Using PCR-based Sanger sequencing, we verified this variant was present in all 10 selected individuals for WES and we confirmed the segregation of this variant with KC in all affected individuals carrying the linkage haplotype except individual III9 (see grey panels in Fig. 1B, Supplemental Table 1). S419 of PPIP5K2 is highly evolutionally conserved from zebrafish to humans (Supplemental Fig. 1), suggesting its potential important function of the PPIP5K2 protein.

We also selected five family members for follow-up analysis with WGS: four KC patients and one control (Fig. 1A, III7, IV2, IV5, IV8, and IV10 with a black circle). Using our established variant filtering pipeline^41^, we verified the non-synonymous mutation in the *PPIP5K2* gene and identified an additional intronic variant rs373951075 (chr5:96,408,333, c.1096–10 G > A, GnomAD MAF 0.00026) in the *PCSK1* gene (proprotein convertase subtilisin/kexin type 1). Using PCR-based Sanger sequencing, we verified this *PCSK1* variant in all available family members which showed complete segregation with KC (Fig. 1C, Supplemental Table 1). Analysis of evolutional conservation only showed invariant nucleotide G in higher mammals and primates (Supplemental Fig. 2). Notably, this variant is located in the ClinVar database with a designation of a variant of unknown significance after being identified in patients with proprotein convertase 1/3 deficiency and monogenic non-syndromic obesity. It is noted that this variant is not present in the mouse/rat genome.

### Sequence variant in *PPIP5K2* in a second family with familial KC

**Figure 2:**
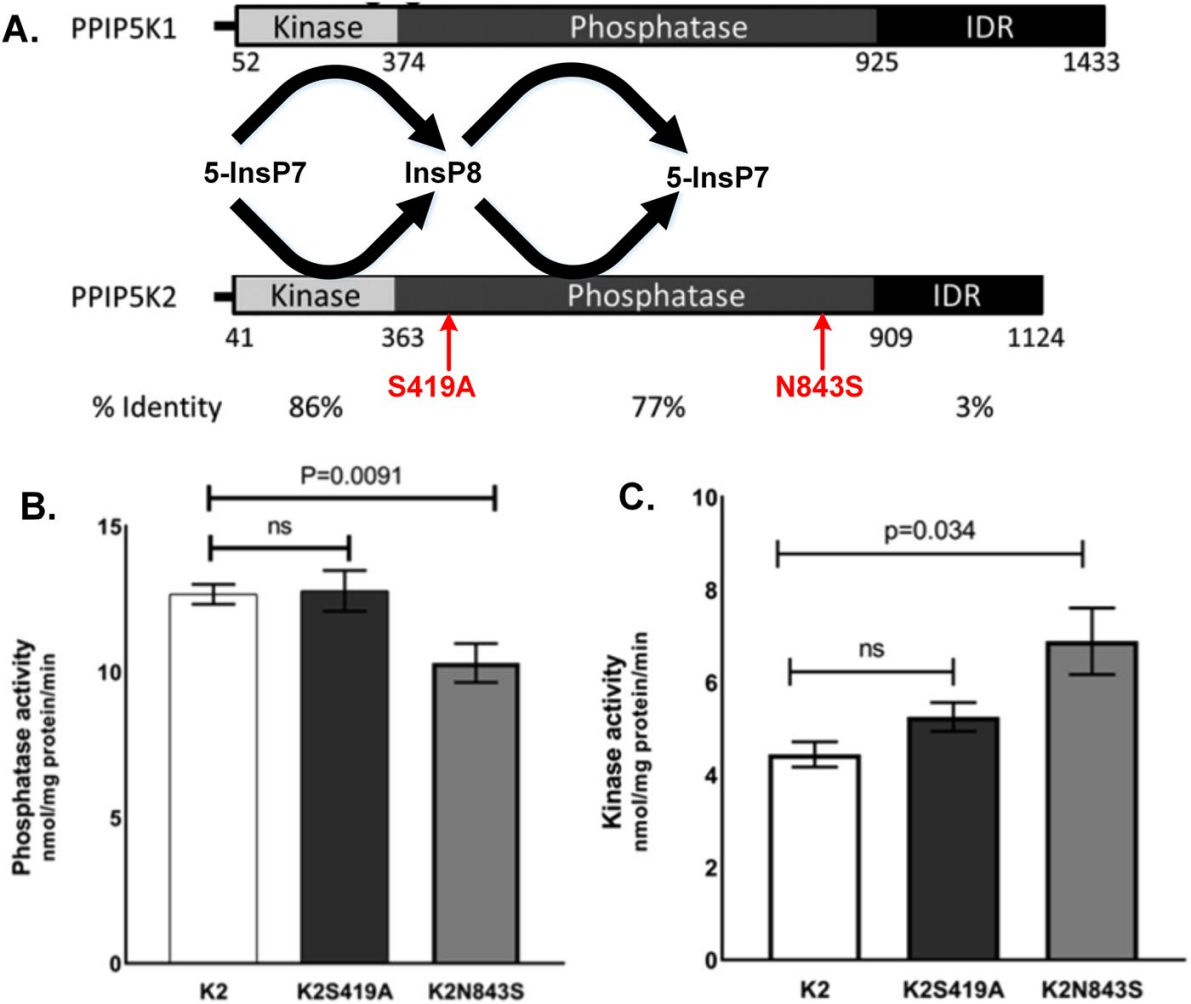
Schematic diagram of PPIP5K1 and PPIP5K2 domains and the *in vitro* biochemical assays for the phosphate and the kinase activities carrying the identified pathogenic variants. (**A**) Diagram of PPIP5K1 and PPIP5K2 functional domains with the identified pathogenic variants. The text with arrows shows our identified pathogenic variants located in the phosphatase domain of PPIP5K2. (**B**) PPIP5K2-phosphatase activity level with the two identified variants. (**C**) PPIP5K2-kinase activity level with the two identified variants. K2 represents hsa-PPIP5K2, K2S419A represents hsa-PPIP5K2 with Serine 419 to Alanine change, and K2N843S represents hsa-PPIP5K2 with Asparagine 843 to Serine change. Each bar represents four separate independent enzymatic assays.

Using WES data from a second KC-affected family with four selected individuals (Fig. 1D, KC: 301 and 303; controls: 201 and 202, indicated by the black arrow), we identified another nonsynonymous variant (rs781831998, c.2528 A > G, p.Asn843Ser, N843S) in the *PPIP5K2* gene with a global MAF of 0.00006 in the GnomAD database. Using PCR-based Sanger sequencing, we successfully verified the mutation and screened for this mutation in other family members (Fig. 1D, Supplemental Table 2). The presence of this missense mutation in other unaffected family members indicates its incomplete penetrance of this missense mutation, similar to the first mutation identified in the four-generation family. Across 100 different vertebrates, the amino acid at this position is either aspartic acid (D) or asparagine (N) (Supplemental Fig. 3). The rare change to serine at this position could impact the protein function of PPIP5K2. Interestingly, both S419A and N843S mutations are located in the phosphatase domain (amino acids 363–909) of the PPIP5K2 protein and could impact the dynamic balance of phosphatase and kinase activities (Fig. 2A). Therefore, we focused our research on the potential role of *PPIP5K2* variants in the pathogenesis of KC.

### Biochemical properties of PPIP5K2 protein carrying the two identified variants

To determine the biochemical effects of both nonsynonymous variants on the PPIP5K2 protein, we used HEK cells as a host for heterologous expression of either wild-type PPIP5K2 protein or each of the S419A or N843S variants; the addition of an N-terminal FLAG tag (FLAG octapeptide or epitope with the sequence DYKDDDDK) assisted protein purification for *in vitro* measurements of the separate kinase and phosphatase activities^42^. These enzymatic assays (n = 4) showed that the N843S variant caused a significant 15% reduction in the PPIP5K2 phosphatase activity (p < 0.05), while the S419A variant did not change the phosphatase activity significantly (p > 0.05) (Fig. 2B). Both variants separately exhibited elevated kinase activities *in vitro*, although this was only statistically significant for the N843S mutation (Fig. 2C). These *in vitro* biochemical assays provide direct evidence that the N843S variant alters the catalytic functions of PPIP5K2. Because of the “futile-cycle” catalyzed by the PPIP5K protein (Fig. 2A), the two separate but reciprocal effects of the N843S variant (inhibition of phosphatase and activation of kinase) can be expected to produce a synergistic enhancement of InsP8 synthesis *in vivo*^40,43^. The significance of S419 to PPIP5K2 functionality remains to be determined; perhaps it has a regulatory role by being a target for covalent modification. This residue lies 25 residues outside the catalytic RHGDRTP motif, within a polyphosphoinositide-binding module^44^.

### Expression of PPIP5K2 in human and mouse ocular tissue and cells

**Figure 3:**
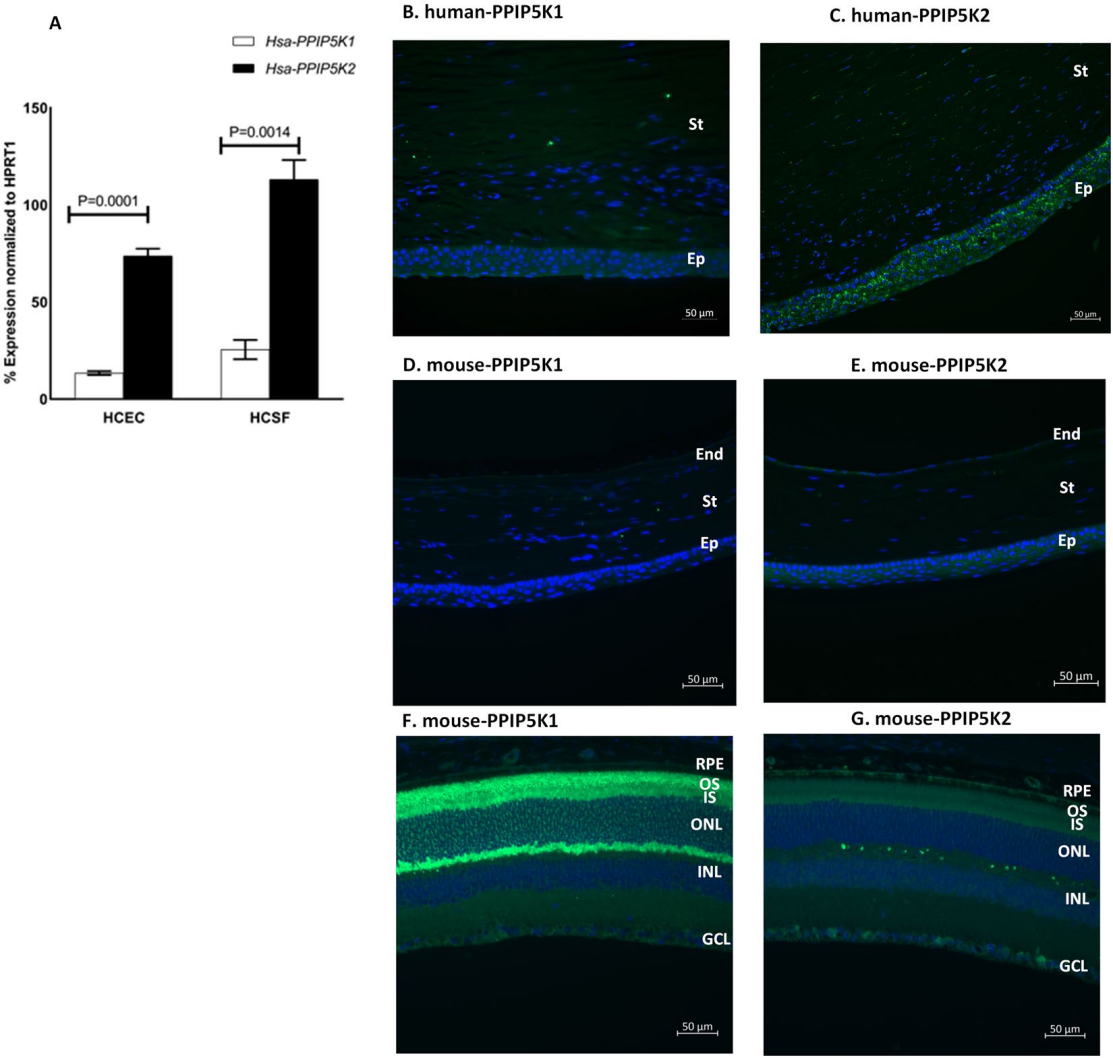
PPIP5K2 expression in corneal cells and tissue. (**A**) Human corneal epithelial cells (HCEC) and human corneal stromal fibroblasts (HCSF) (n = 3) showed higher expression of *PPIP5K2* transcripts than *PPIP5K1*. (**B**) PPIP5K1 (green) and (**C**) PPIP5K2 (green) protein expression in human cornea. (**D**) PPIP5K1 (green) and (E) PPIP5K2 (green) protein expression in mouse cornea. (**F**) PPIP5K1 and (**G**) PPIP5K2 protein expression in mouse retina. Error bars represent standard error of the mean. Green fluorescence represents the target protein, blue fluorescence represents DAPI (4′,6-diamidino-2-phenylindole). Abbreviations: Ep for epithelium, St for stroma, End for endothelium, RPE for retinal pigment epithelium, OS for outer segment, IS for inner segment, ONL for outer nuclear layer, INL for inner nuclear layer, and GCL for ganglion cell layer.

We used ddPCR (droplet digital PCR) assays to examine the expression of *PPIP5K2* and its paralog *PPIP5K1* in primary human corneal epithelial cells (HCEC) (n = 3), and also in primary human corneal stromal fibroblast (HCSF) cells (n = 3). We found that *PPIP5K2* mRNA showed 5-fold higher expression than *PPIP5K1* mRNA in human HCEC and HCSF cells (Fig. 3A). Immunofluorescence staining in human and mouse corneas and mouse retinas demonstrated an exclusive high expression of PPIP5K2 protein in both human (Fig. 3B,C) and mouse corneas (Fig. 3D,E), while PPIP5K1 showed higher expression in mouse retina (Fig. 3F,G). The unique expression of PPIP5K2 but not PPIP5K1 in human and mouse corneas confirms the potential functional significance of PPIP5K2 in corneal physiological processes.

### Corneal pathological phenotypes in *Ppip5k2*^*+/K^*^ & *Ppip5k2*^*K^/K^*^ mouse model

**Figure 4:**
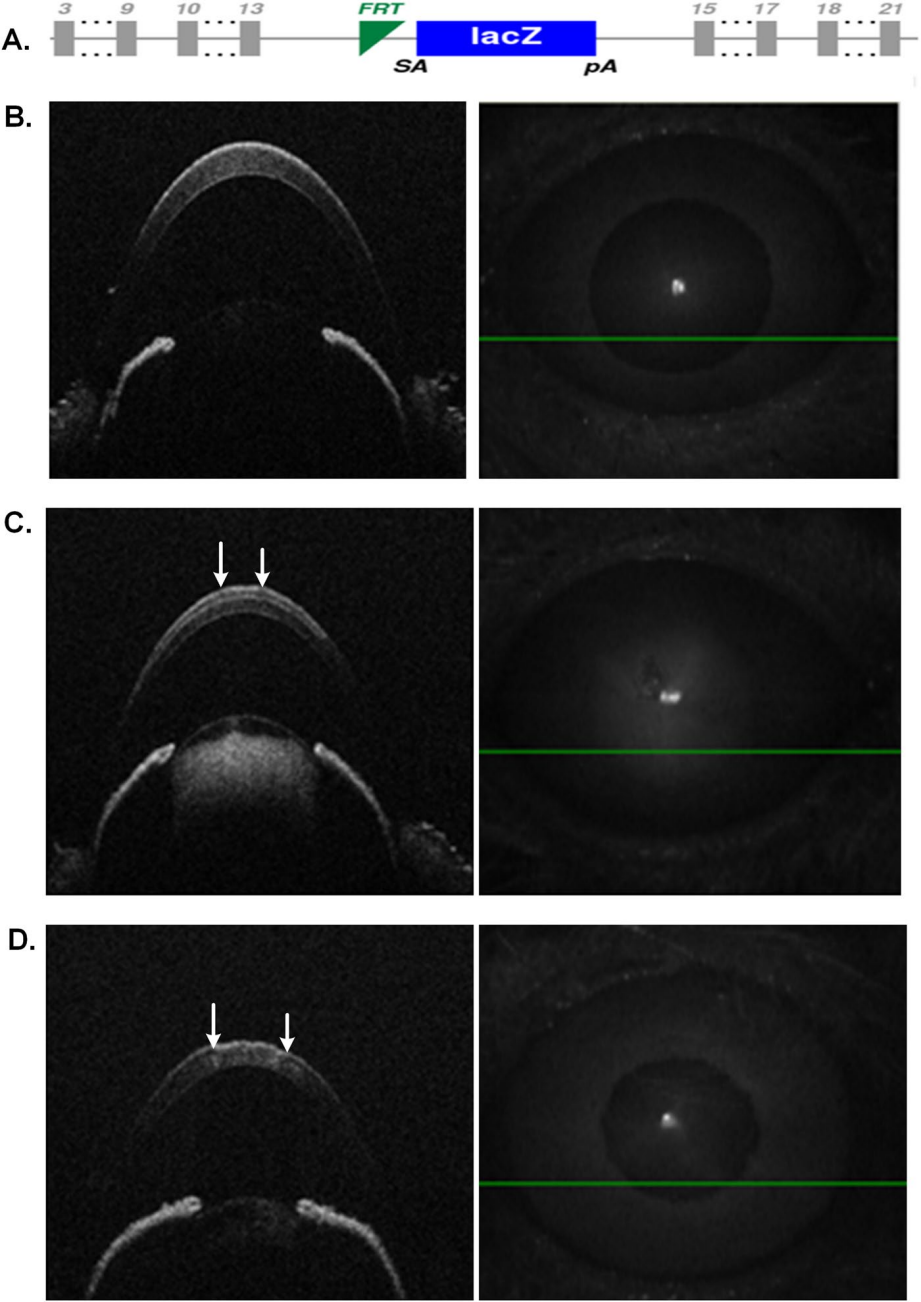
Development and Optical Coherence Tomography characterization of the *Ppip5k2* gene-trap mouse model. (**A**) Allele map and design of the *Ppip5k2*^*K^/K^*^ mouse model; (**B–D**) SD-OCT images of the anterior chamber of the eye. (**B**) a *Ppip5k2*^+*/*+^ wild-type mouse; (**C**) a *Ppip5k2*^+/*K^*^ heterozygous mouse; (**D**) a *Ppip5k2*^*K^/K^*^ homozygous mouse. The white arrows indicate the presence of irregular anterior surface in the cornea.

In order to model the elevated kinase and reduced phosphatase activity in PPIP5K2, we obtained a *Ppip5k2*-gene-trap mouse B6N(Cg)-*Ppip5k2*^*tm1b(EUCOMM)Wtsi*^*/J* from the Jackson Laboratory. A beta-galactosidase containing cassette with an upstream 3′ splice site and a downstream transcriptional termination sequence with a poly(A) tail was inserted as a reporter allele while exon 14 was removed (Fig. 4A). This mouse carried a truncated Ppip5k2 protein containing the full kinase domain and a truncated (limited to the first 82 residues) phosphatase domain, which eliminates phosphatase activity and increases kinase activity^42^. The allele has previously been designated as *Ppip5k2*^*K^*^ to emphasize its elevated kinase activity^42^. We used the littermate wild-types as our controls. The colony was maintained by crossing *Ppip5k2*^+*/K^*^ to *Ppip5k2*^+*/K^*^ or *Ppip5k2*+^*/K^*^ to *Ppip5k2*^+*/*+^. This age of 3-month was chosen because murine corneal epithelium and corneal stroma thickness stabilize after 2 months of age^45^. In addition, this age is equivalent to the prevalent teenage onset of KC in human patients^46^.

**Figure 5:**
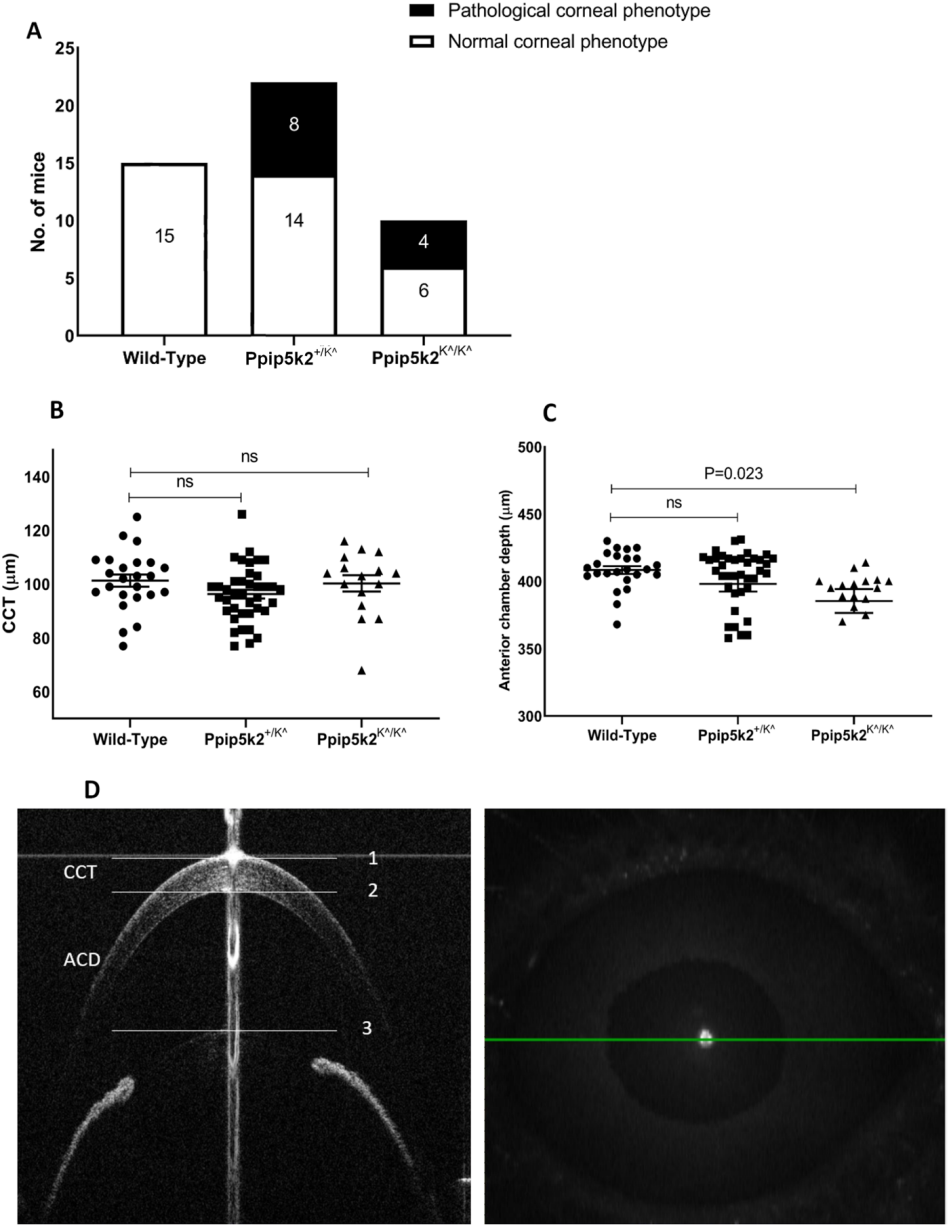
SD-OCT-based mouse corneal measurements in three different mouse groups: *Ppip5k2*^+*/*+^ wild-type, *Ppip5k2*^+*/K^*^ heterozygous, and *Ppip5k2*^*K^/K^*^ homozygous mice. (**A**) Percentage of mice with irregular anterior corneal surfaces found with SD-OCT scanning among the three different groups. (**B**) Central corneal thickness (CCT) measurements among three groups. (**C**) Anterior chamber depth (ACD) among three groups. (**D**) SD-OCT image of a *Ppip5k2*^+*/*+^ mouse representing the method to calculate CCT and ACD.

**Figure 6:**
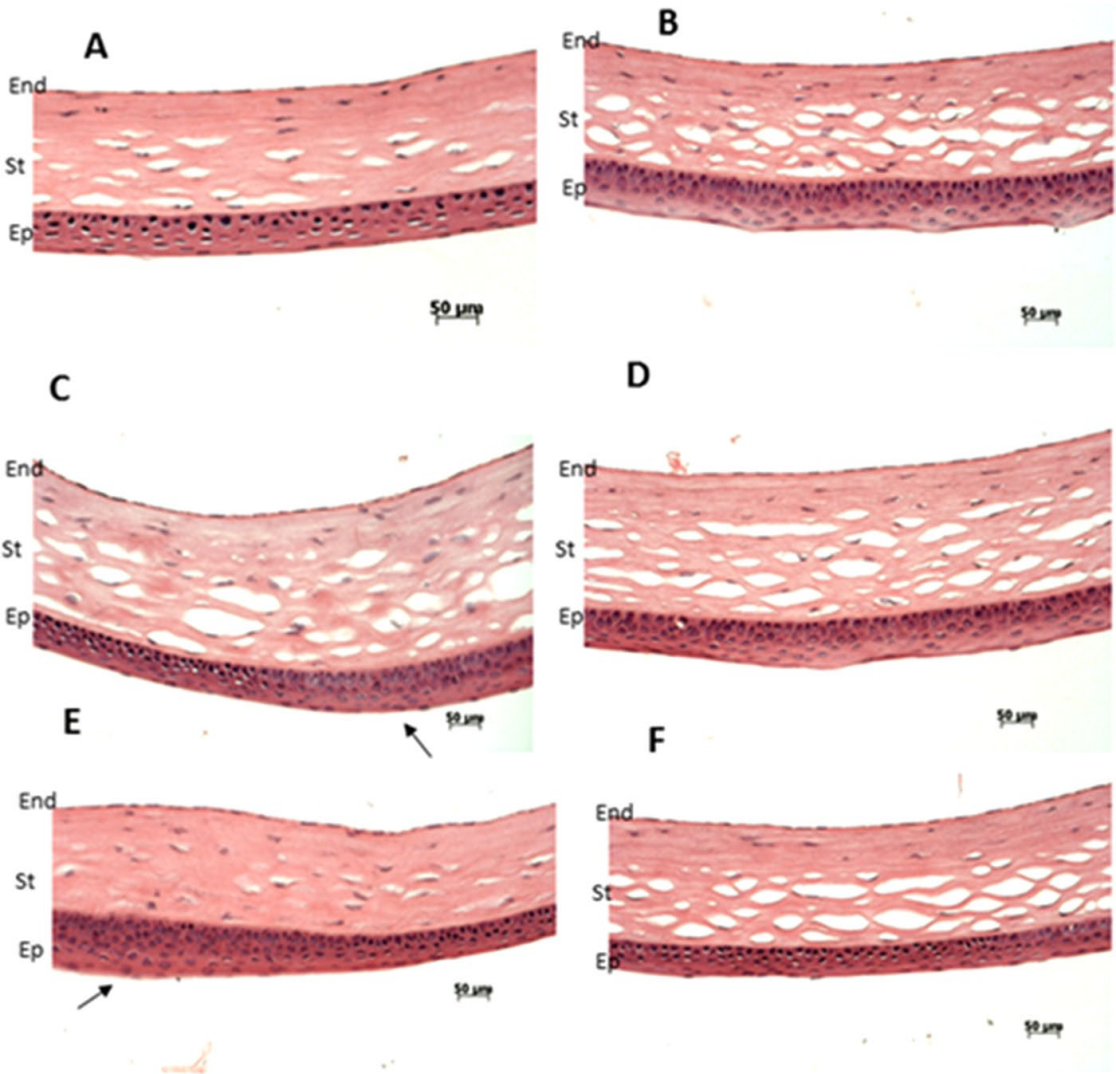
H&E staining of the mouse corneas. Normal epithelium was observed in wild-type *Ppip5k2*^+*/*+^ littermate controls (**A**,**B**). Thickened corneal epithelium layer was observed in some *Ppip5k2*^+*/K^*^ heterozygous **(C)** and *Ppip5k2*^*K^/K^*^ homozygous **(E)** mouse corneas. These corneas (**C**,**E**) were found to have irregular anterior corneal surfaces using SD-OCT scanning. Normal corneal epithelium thickness was noticed in other *Ppip5k2*^+*/K^*^ heterozygous (**D**) and *Ppip5k2*^*K^/K^*^ homozygous (**F**) mouse corneas. These corneas (**D**,**F**) were found to have no irregular anterior corneal surfaces using OCT scanning at 3 months of age. The black arrows showed the thickened epithelium in panels C and E. Abbreviations: Ep for epithelium, St for stroma, and End for endothelium of the mouse cornea.

**Figure 7:**
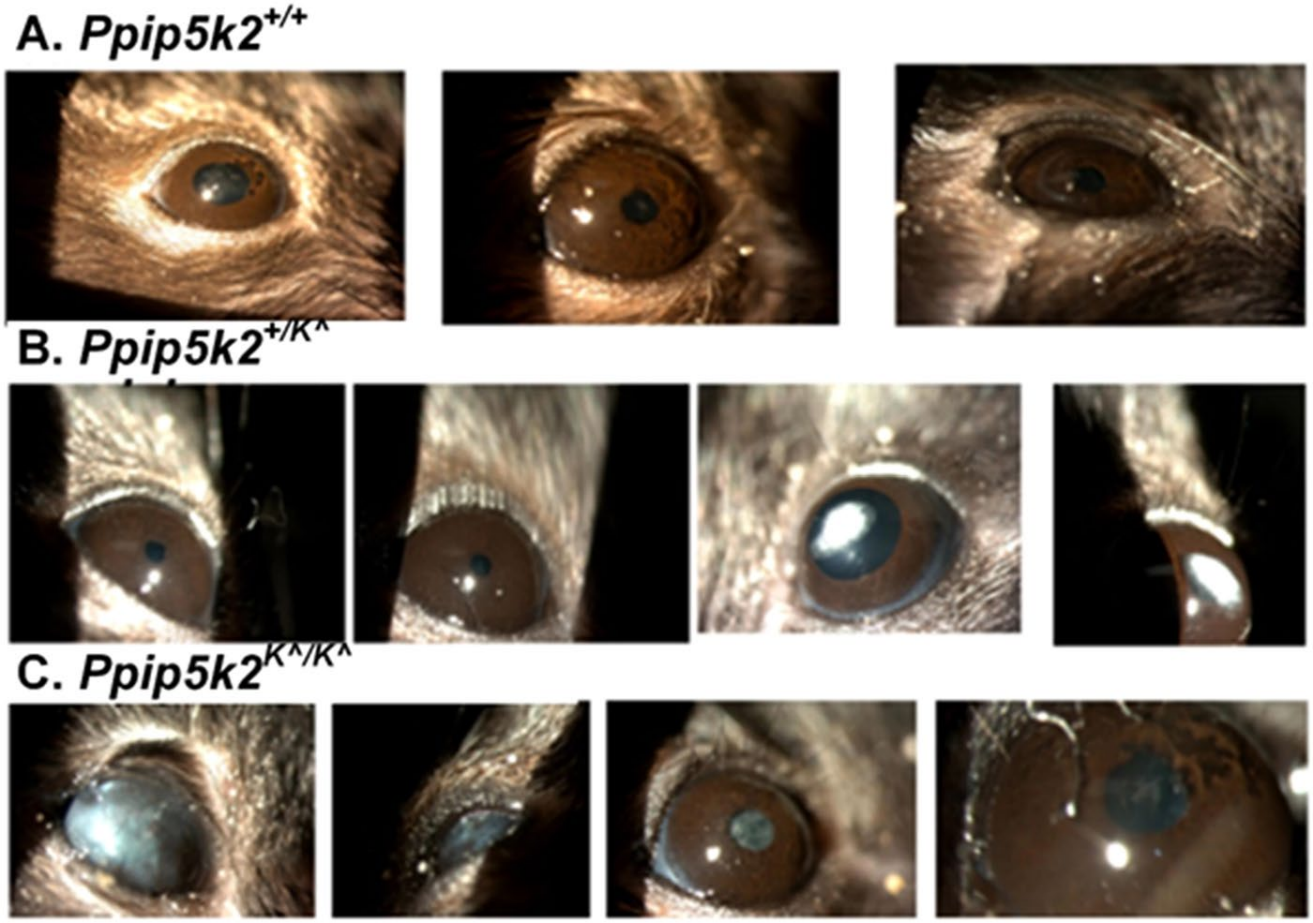
Slit lamp biomicroscopic examination of mouse cornea. Images of mouse cornea at 3 months of age with wild type *Ppip5k2*^+*/*+^ (**A**), heterozygous *Ppip5k2*^+*/K^*^ (**B**), and homozygous ^*Ppip5k2K^/K^*^ mice (**C**).

We used Spectral Domain Optical Coherence Tomography (SD-OCT) scanning, histological staining, and slit lamp bio-microscopy to visualize the anterior segment, including the cornea. We observed irregularities on the anterior corneal surface in the *Ppip5k2*^+*/K^*^ (Fig. 4C) and *Ppip5k2*^*K^/K^*^ (Fig. 4D) mice at three months of age compared to their littermate *Ppip5k2*^+*/*+^ controls (Fig. 4B). Approximately 40% of the *Ppip5k2*^+*/K^*^ and *Ppip5k2*^*K^/K^*^ mice showed significantly corneal surface irregularities of the anterior surface (Fig. 5A), indicating potential incomplete penetrance, similar to that of KC in human patients. Central cornea thickness (CCT) measurements did not change significantly across the three different genotypes (Fig. 5B), while the anterior chamber depth was significantly reduced in the *Ppip5k2*^*K^/K^*^ mice (Fig. 5C). H&E (Hematoxylin & Eosin) staining of mouse corneal sections revealed that these surface irregularities primarily manifested as thickened epithelium (Fig. 6). Slit lamp examination revealed corneal opacity of mouse corneas in the *Ppip5k2*^+*/K^*^ and *Ppip5k2*^*K^/K^*^ mice (Fig. 7).

**Figure 8:**
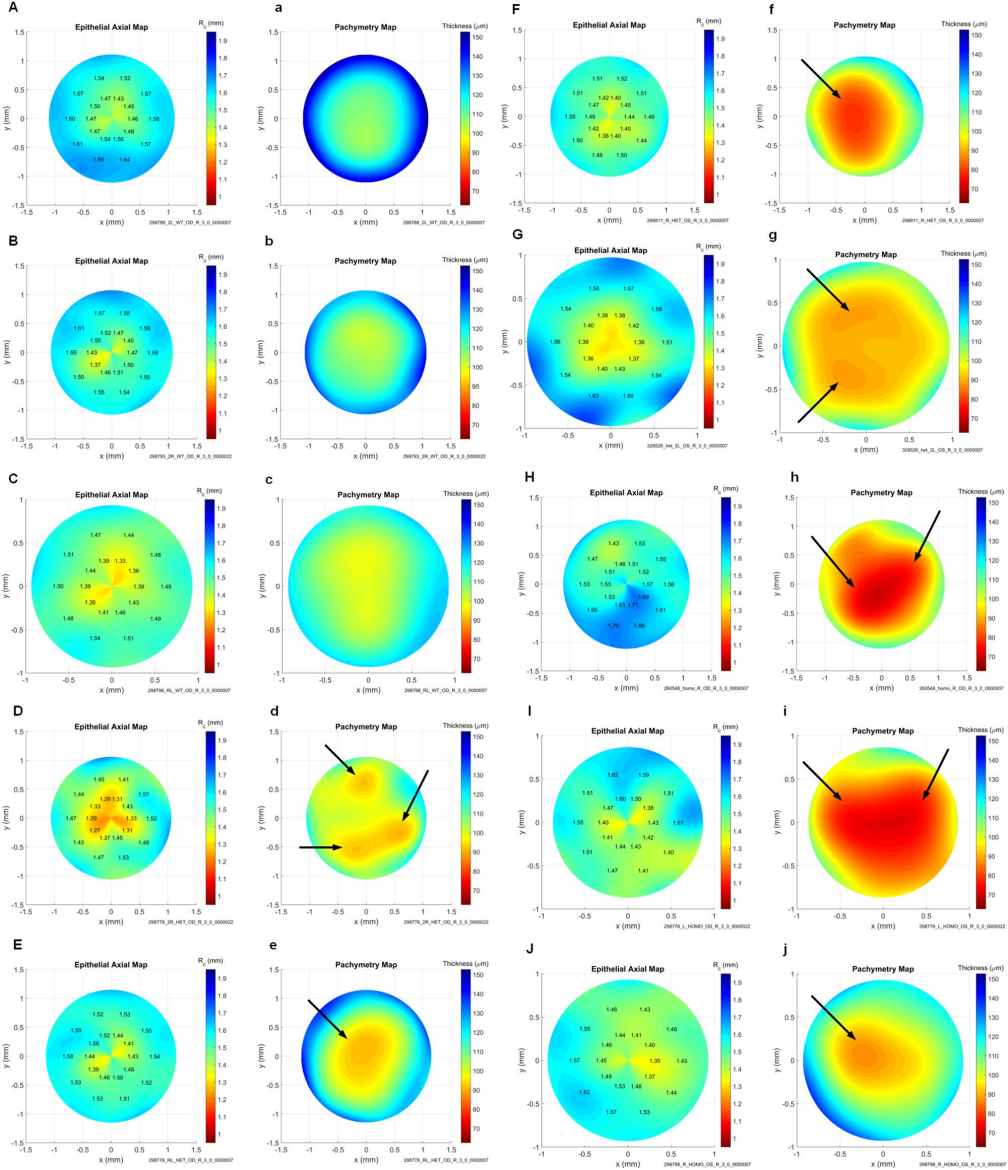
Mouse corneal curvature and corneal thickness mapping. The uppercase letter represents curvature map (epithelial axial map), while the lowercase letter represents the thickness map (pachymetry). Panels (A–C), (**a–c**) for wild-type *Ppip5k2*^+/+^ mice, (D–G**)**, (**d–g**) for the *Ppip5k2*^+/*K^*^ mice, and (**H–J**), (**h–j)** for the *Ppip5k2*^*K^/K^*^ mice. All mice were at 3–4 months of age. The focally abnormal thin cornea regions were marked with black arrows.

Corneal topography/tomography and pachymetry mapping have been integrated into the clinical examination and diagnosis of human KC patients. However, a similar approach in mouse corneas was not implemented prior to this current study. We used calibrated mouse corneal OCT scans and generated mouse cornea tomography and pachymetry maps of *Ppip5k2*^+*/*+^ (n = 3), *Ppip5k2*^+*/K^*^ (n = 4), and *Ppip5k2*^*K^/K^*^ (n = 3) mice at 3-month of age (Fig. 8). OCT-derived pachymetry mapping exhibited significant diffuse corneal thinning in the *Ppip5k2*^+*/K^*^ (Fig. 8d–g) and *Ppip5k2*^*K^/K^*^ mice (Fig. 8h–j) versus their littermate *Ppip5k2*^+*/*+^ controls (Fig. 8a–c). The anterior curvature map of mouse corneas (Epithelial Axial Map) showed localized curvature perturbations in the mutant mice (Fig. 8). The irregularities of the mouse anterior corneal surface and localized abnormal corneal thinning in the *Ppip5k2*^+/*K^*^ and *Ppip5k2*^*K^/K^*^ mice clearly indicate the critical role of Ppip5k2 in maintaining the physiological function of the mouse cornea, consistent with the potentially important role of PPIP5K2 in the pathogenesis of KC in human patients. However, the slightly more shallow anterior chamber in the *Ppip5k2*^*K^/K^*^ mice was unexpected, since in humans KC has frequently been associated with a deeper anterior chamber^47–49^. On the other hand, deeper anterior chamber depth by itself has limited clinical utility in screening for KC^50^.

### Visual function changes in *Ppip5k2*^*+/K^*^ & *Ppip5k2*^*K^/K^*^ mouse model

Assessment of visual function using the OptoMotry system to evaluate the mouse optokinetic response did not show any significant differences in the visual acuity of wild-type *Ppip5k2*^+*/*+^ (n = 7), *Ppip5k2*^+*/K^*^ (n = 5), or *Ppip5k2*^*K^/K^*^ (n = 3) mice (data not shown). Contrast sensitivity of the wild-type mice was 8.5 which was higher than the values of 4.4 and 3.6 for the *Ppip5k2*^+*/K^*^ and *Ppip5k2*^*K^/K^*^ mice, respectively (data not shown). However, these differences were not statistically significant.

## Discussion

We have successfully identified two novel potentially pathogenic variants in the phosphatase domain of the *PPIP5K2* gene in two autosomal dominant multiplex KC families. Variant S419A is identified in a four-generation US family with European ancestry, and variant N843S is present in an unrelated two-generation family with European ancestry. Our *in vitro* studies indicate the N843S variant separately decreases InsP8 phosphatase activity and elevates InsP7 kinase activity (Fig. 2), likely resulting in a synergistic activation of InsP8 synthesis *in vivo*^40,43^. This is the first study to suggest the role of *PPIP5K2* coding variants in the genetic etiology of keratoconus. Our mouse model with no phosphatase and hyperkinase activity of Ppip5k2 recapitulates the impact of elevated InsP8 synthesis *in vivo*^42^ and, moreover, demonstrated abnormal localized corneal curvature and thinning, as well as epithelial histological changes. Our study is the first to successfully apply OCT-based corneal tomography and pachymetry mapping in mouse models to study its relationship with KC. The animal model could be used to further characterize the pathogenesis of KC in the future.

PPIP5K2 and its products (InsP7 and InsP8) play a vital role in the inositol phosphate signaling pathway via regulation of energy homeostasis, phosphate (Pi) sensing, and immune responses^38,51,52^.

*PPIP5K2* and its paralog *PPIP5K1* encode dual functional enzymes which each contain separate kinase and phosphatase domains. The kinase activity converts 5-InsP7 to 1,5-InsP8 while the phosphatase dephosphorylates 1,5-InsP8 to 5-InsP7. To investigate the relative expression of PPIP5K1 and PPIP5K2 in the cornea, we measured their mRNA and protein levels in human corneal cells and tissues. Our data showed a significantly higher expression level of PPIP5K2 in HCEC and HCSF than PPIP5K1. Moreover, immunohistochemistry of human and mouse cornea demonstrated a higher expression of PPIP5K2 than PPIP5K1. These data reveal that PPIP5K2 is the dominant enzyme in the cornea suggesting an important role of this enzyme in the cornea. Interestingly, PPIP5K1 demonstrated a markedly high level of expression in the mouse retina indicating a potential specific function of PPIP5K1 in this particular tissue.

To study the impact of the identified pathogenic variants on the PPIP5K2 catalytic activity, we introduced the point mutations in plasmid constructs encoding the enzyme and a FLAG epitope tag; these constructs were transiently transfected into HEK293 cells, and then the recombinant proteins were purified and assayed for both kinase and phosphatase activities. Interestingly, the p.Asn843Ser point mutation reduced phosphatase activity and increased kinase activity. These two separate but complementary effects of the N843S variant (inhibition of phosphatase and activation of kinase) can be expected to produce a synergistic enhancement of InsP8 synthesis *in vivo*^40,43^. On the other hand, the p.Ser419Ala mutation did not show a significant disturbance in PPIP5K2 enzymatic activity. Other regulatory factors might influence the effect of the p.Ser419Ala mutation such as inorganic phosphate and glucose, or binding partners^40^, but we have not yet studied this possibility.

The impact of N843S variant *in vitro* (a decreased phosphatase activity and a separate activation of kinase activity) is reminiscent of a previous study^42^, that described the impact of an R837H variant of PPIP5K2. Both R837 and N843 could influence phosphatase activity by virtue of being only slightly C-terminal to His828 (which is part of the catalytic H[D/I/V/A] dipeptide)^53^. The influence of the N843S and R837H variants upon the PPIP5K2 kinase activity, which resides in a separate domain, would presumably involve some form of long-range allosteric coupling.

The availability of multiple inbred mouse strains and their physiological similarity to humans make mouse models attractive to study human genetic disorders^54^. Accordingly, to recapitulate genetically-based increased InsP8 synthesis *in vivo*, we used a *Ppip5k2* gene trapped ‘hyperkinase’ mouse model with beta-galactosidase containing cassette to replace exon 14. We examined the cornea anterior curvature from three groups of mice: *Ppip5k2*^+*/K^*^, *Ppip5k2*^*K^/K^*^ and their littermate wild type *Ppip5k2*^+/+^ controls at 3–4 months of age.

An important feature of our study has been to enhance the human-health significance of our findings, by application of diagnostic tools routinely used for diagnosis of corneal abnormalities by corneal specialists such as SD-OCT, slit lamp, and corneal histology to assess mouse corneal structural changes. Slit lamp examination revealed varying degrees of corneal opacification and a shallow anterior chamber in the *Ppip5k2*^+*/K^*^ and *Ppip5k2*^*K^/K^*^ mice versus their littermate controls. A slight reduction in anterior chamber depth was also observed by SD-OCT, albeit only in the *Ppip5k2*^*K^/K^*^ mice. Interestingly, human KC patients often have a deeper anterior chamber^47–49^. In that sense, our data mirror some concerns in the literature of the clinical utility of anterior chamber depth in screening for KC^50^. However, we do note the relatively small sample size of the homozygous *Ppip5k2*^*K^/K^*^ mice (Fig. 5C).

Among the three different groups (wild type, *Ppip5k2*^+*/K^*^, and *Ppip5k2*^*K^/K^*^), approximately 40% of the mutant mice exhibited the abnormal anterior corneal curvature, indicating incomplete penetrance. High quality OCT-based anterior curvature and thickness mapping clearly indicates abnormally thin localized corneal regions in the *Ppip5k2*^+*/K^*^ mice, whereas the *Ppip5k2*^*K^/K^*^ mice presented with more diffuse corneal changes. Nevertheless, our study is the first to successfully apply OCT-based corneal tomography and pachymetry mapping in mouse models to study its relationship with KC. Additional histological staining showed pronounced epithelial abnormalities.

The corneal opacities, abnormal cornea structure and surface irregularities observed in the *Ppip5k2*^+*/K^*^ and *Ppip5k2*^*K^/K^*^ mice have the potential to impair visual function by interfering with the amount of light reaching the retina. For *Ppip5k2*^+/*K^*^ and *Ppip5k2*^*K^/K^*^ mice visual acuity measured as the spatial frequency threshold at 100% contrast was similar to wild-type mice. The *Ppip5k2*^+*/K^*^ and *Ppip5k2*^*K^/K^*^ mutant mice did, however, have reduced contrast sensitivity compared to wild-type mice. Corneal abnormalities may well mimic conditions such as low light or glare which can reduce the mouse’s ability to detect differences in contrast.

In addition to the pathogenic variants in the *PPIP5K2* gene, WGS revealed an intronic variant within the *PCSK1* gene (rs373951075, c.1096-10 G > A) in the four-generation family. This variant is located in the narrow region identified by both linkage and association analysis of this specific family with complete segregation of KC haplotype. Interestingly, heterozygous *Ppip5k2*^+*/K^*^ mice (Fig. 8) showed localized corneal thinning patterns resembling KC-associated clinical phenotype while homozygous *Ppip5k2*^*K^/K^*^ mice had a more progressed diffuse pattern of corneal thinning resembling that of keratoglobus-related phenotype. These different phenotypes suggest that other genetic factors such as the intronic variant in *PCSK1* may work together with PPIP5K2 to cause specific clinical features associated with KC.

Based on our bioinformatics analysis, this variant of *PCSK1* is located in a consensus sequence that binds with JunD, an AP-1 transcription factor subunit. The consensus sequence includes a cAMP-responsive element (CRE) motif to which JunD binds and forms heterodimers with the ATF (activating transcription factor) family members^55,56^. JunD acts as a transcriptional activator or repressor of specific target genes to regulate diverse vital processes including cellular proliferation, differentiation and oxidative stress^57^. Sequence change at this specific location may lead to altered JunD binding and thus potentially affect the hormone processing activity of PCSK1. Recent studies have suggested the potential role of hormone-related factors such as prolactin and 17β-estradiol in the pathogenesis of KC^13,46,58–60^. It may also be interesting to study the potential interaction of this intronic variant and *PPIP5K2* coding variants in the future.

It may also be relevant that *PPIP5K2* is a predicted target gene of transcription factor PRDM5 (PR/SET Domain 5) through ChIP-Seq (chromatin immunoprecipitation sequencing) experiment^61^. Homozygous mutations of *PRDM5* have been identified in human patients with brittle cornea syndrome^61–64^. *PRDM5* mutations are considered a strong predisposing factor for the development of KC in several families^64^. It will be important to confirm the potential functional connection between PRDM5 and PPIP5K2 in human and mouse corneas.

It has been recognized that genetic susceptibility to KC could be modified by environmental, hormonal, and biomechanical factors as well as inflammation factors^13,65^. In spite of the large number of identified linkage loci, genetic mutations have been reported in only a limited number of genes including *MIR184* and *DOCK9*^7,18,27,29,30,32,66^. This could be due to complex inheritance patterns such as digenic inheritance^67^. The penetrance of genetic mutations could be affected by mechanical/environmental factors such as eye rubbing, UV exposure, atopic conditions, and the sensitivity of clinical detection of sub-clinical corneal phenotypes in patients, as well^3,7,65^. The incomplete penetrance and co-segregation of genetic mutations have been noted in our familial study. Therefore, *in vitro* and *in vivo* functional studies are critical to characterize the identified mutations.

In conclusion, we have identified two novel variants located in the phosphatase domain of the *PPIP5K2* gene in two US families with familial KC and one potentially functional variant in the *PCSK1* gene. Our *in vitro* catalytic assays and *in vivo* mouse model strongly suggest a role for PPIP5K2 in the pathogenesis of KC. It is the first time to integrate mouse corneal tomography and pachymetry mapping in mouse corneal phenotyping. However, further studies will be valuable to investigate the exact effect of the identified variants in the cornea. This could be accomplished by using a CRISPR-mediated knock-in mouse model for the identified variants.

## Methods

### Human subjects and DNA Isolation

The Institutional Review Board at the Cedars Sinai Medical Center approved the research protocol. All patients and family members were informed about the study and consented using written informed consent. DNA was extracted from Epstein-Barr virus (EBV) - transformed lymphoblast cells of all family members using Puregene kit^24,37^. DNA Samples >10 kb in size with no or little degradation were selected for WES and WGS. All methods were performed in accordance with the relevant guidelines and regulations.

### Whole exome and whole genome sequencing

For WES, fourteen high quality DNA samples were enriched with Roche NimbleGen SeqCap EZ Exome Library v3.0 followed up by 100 bp paired-end sequencing as previously described^41^. The bioinformatics analysis was performed by the Bioinformatics Core at Duke University Medical Center as previously described^41^. Briefly, sequencing reads were aligned to human reference genome NCBI build 37 with the BWA (Burrows-Wheeler Alignment) algorithm^68^. Potential PCR duplicate reads were removed with Picard and variants were called with GATK (Genome Analysis Tool Kit), following the Broad Institute’s Best Practices Workflow^69–71^. The called variants were annotated for their functional impact using SnpEff (SNP effect) with annotations from Ensembl^72^. All the DNA samples were sequenced with at least 75x average coverage for all targeted regions.

Five individual DNA samples were selected for additional WGS analysis (Fig. 1A) with paired-end 150 bp sequencing using Illumina HiSeq XTEN sequencer with >30X average coverage at New York Genome Center (NYGC). Bioinformatics analysis was performed using the standard automated high-performance pipeline at NYGC. Reads were aligned to the GRCh37 human reference genome using the Burrows-Wheeler Aligner (BWA-MEM v0.78)^68^ and processed using the best-practices pipeline that includes marking of duplicate reads by using Picard, realignment around indels, and base recalibration via GATK v3.2.2^70^. To improve variant call accuracy, five single-sample GVCF files were jointly genotyped using GATK GenotypeGVCFs to generate a multi-sample VCF (variant call format) file. Variant annotations were done using SnpEff^72^, VCFtools^73^, and NYGC in-house custom software.

### Sequence variant filtering pipeline

We applied a specific sequence analysis pipeline in a combination with RNA-Seq-based gene expression data from 9 unaffected human corneas to filter and prioritize all the variants (Supplemental Fig. 4). We used the SNP & Variation Suite (SVS) version 8.0 software from Golden Helix for our analysis. We filtered the variants using the following criteria: 1) good sequencing quality and high read depth (>10–20); 2) absence in the unaffected controls without linkage haplotype; 3) MAF ≤ 0.01 in publicly available variant databases (The Exome Aggregation Consortium (ExAC), NHLBI GO Exome Sequencing Project (ESP), Genome Aggregation Database (gnomAD), and 1000 Genomes); 4) coding non-synonymous variants; 5) heterozygous in the affected patients; 6) located in genes with expression in human cornea; and 7) located within/adjacent the linkage locus chr5q14.3–21.1. We applied a similar filtering pipeline for the WGS data to prioritize the variants (Supplemental Fig. 5).

### *In vitro* functional assays for PPIP5K2

Plasmids hosting human *PPIP5K2* (NM_001345875) were constructed as previously described^39,40^. Individual variants S419A or N843S were constructed using the Q5 site-directed mutagenesis kit (New England Biolabs). The paired primers were designed as follows (mutagenic nucleotide is lower case): S419A forward: TGGATATAAAgCAGGGAAATTAAAAC, and the reverse: TCACACTTTTCAAAAAGATCAAAAAATTTC; N843S forward: GCCTTATGCAgTGAATCAAAG, and the reverse: ACCATAGCGAAGAATAGAC. HEK293 cells were transiently transfected with plasmids containing FLAG-tagged PPIP5K2, PPIP5K2 (p.S419A) or PPIP5K2 (p.N843S) using Lipofectamine 3000 (Life Technologies). Cells were harvested and lysed 16–20 hours after transfection. The FLAG-tagged PPIP5K2 proteins were immunopurified using FLAG M2 affinity gel (MilliporeSigma) and analyzed by SDS-PAGE and stained with Coomassie Blue.

The enzymatic assays of PPIP5K2 phosphatase and kinase activities were performed as previously described^39^. Briefly, PPIP5K2 phosphatase activities were measured at 37 °C for 30 min incubations. The activities were calculated by how much InsP8 hydrolyzed to 5-InsP7, which was quantified by HPLC. Kinase activities were measured at 37 °C for 1 h incubations. The reaction was quenched and the conversion of 5-InsP7 to InsP8 was determined by HPLC.

### Culture of primary human corneal epithelial cells and stromal fibroblasts

Human donor eyes were received from the Georgia Eye Bank (Atlanta, GA, USA) within 24 hours after death. Primary HCEC and HCSF were isolated from the donors’ eyes according to the established protocols^74,75^. HCEC were cultured in high glucose DMEM (Dulbecco’s modified eagle medium, Gibco), 10 ng/mL human recombinant EGF (epidermal growth factor), 10% fetal bovine serum, 1% insulin-transferrin-selenium-100× (Gibco), and 40 µg/mL gentamycin (Sigma). The HCSF were cultured similarly as HCEC but without human recombinant EGF.

### *PPIP5K1* and *PPIP5K2* expression with ddPCR

Specific EvaGreen-based ddPCR assays for *PPIP5K1* (dHsaEG5000994) and *PPIP5K2 (*dHsaEG5018506) were obtained from Bio-Rad Laboratories, Inc., to measure the expression of *PPIP5K1* and *PPIP5K2* in primary HCEC and HCSF as previously described^76,77^. We integrated two internal reference genes (*GAPDH* - glyceraldehyde 3-phosphate dehydrogenase and *HPRT1 -* hypoxanthine phosphoribosyltransferase 1) to be quantified in the samples for normalization purpose. Both showed similar consistent expression among all samples. For figure purposes we normalized with *HPRT1*^78^.

### Mouse husbandry

We obtained breeding pairs of heterozygous B6N (Cg)-*Ppip5k2*^*tm1b (EUCOMM)Wtsi*^*/J* mice from the Jackson Laboratory (Fig. 4A) (Bar Harbor, ME). This mouse model has no phosphatase activity, but elevated kinase activity (designated as *Ppip5k2*^*K^*^)^42^. A beta-galactosidase containing cassette with an upstream 3′ splice site and a downstream transcriptional termination sequence with a poly(A) tail was inserted as a reporter allele to replace exon 14. Mice were bred and maintained according to the guidelines described in the Association for Research in Vision and Ophthalmology Statement for the Use of Animals in Vision and Ophthalmic Research and the Augusta University animal handling guidelines. The mouse protocol was approved by the Institutional Animal Care and Use Committee (IACUC) at Augusta University. Following the Jackson Laboratory protocol, mouse genotype was assessed using PCR with the recommended conditions.

### *In vivo* mouse eye examination

For slit lamp and SD-OCT examination live mice were anesthetized using a rodent anesthesia cocktail as previously described^79–81^. GenTeal Lubricant Eye Gel (Alcon, Ft. Worth, TX, USA) was applied after anesthesia until the examination started. Systane lubricant eye drops from Alcon were applied throughout the eye examination to avoid cornea dryness.

The cornea and anterior chamber of anesthetized mouse were examined with a slit lamp (SL-D7; Topcon, Tokyo, Japan), and images were documented with a digital camera (D100; Nikon, Tokyo, Japan). We also used SD-OCT to visualize the anterior segments in mice^45,82^. The anterior chamber structure and corneal curvature of the anesthetized mice were examined using Bioptigen Spectral-Domain Ophthalmic Imaging System (Envisu R2200; Bioptigen)^83^. Briefly, we used a 12 mm telecentric probe and set the reference arm at position 245. Three scans were performed for each eye, producing one rectangular and two radial scans. The central corneal thickness and the anterior depth were assessed using the default DIVERS software (Fig. 4D**)**.

### Corneal curvature and corneal thickness mapping

Corneal biometric parameters including corneal curvature, topography, and pachymetry were measured from the corneal OCT images using methods previously described for human corneal OCT images adapted for mouse corneal OCT images^84,85^.

From the corneal surface data, pachymetry (corneal thickness) maps were created by direct z-axis subtraction of the epithelial and endothelial layers. Curvature maps were created by calculating local epithelial curvatures based on formulas outlined in the ANSI Z80.23-2007 standards for human corneal topography^86^. Instantaneous curvatures were calculated along meridians across the nominal optical zone. A radius of curvature (R_c_) to describe the average curvature of each specific cornea was calculated by fitting the anterior corneal surface to a sphere using least squares fitting.

### Histology and immunohistochemistry in human and mouse corneas

Postmortem human corneas were obtained from North Carolina Eye Bank. Whole mouse eyes were removed immediately after euthanizing the animals. Human cornea or mouse eyes were fixed in Davidson’s fixative for <24hrs, transferred to 70% ethanol. Mouse corneas were processed for H&E staining at the AU Electron Microscopy and Histology Core. Human corneas were processed at the Georgia Esoteric and Molecular (GEM) Laboratory for H&E staining.

For immunohistochemistry, mouse or human cornea sections were rehydrated and incubated with rabbit anti-PPIP5K2 (Abcam, ab204374, 1:100), or anti-PPIP5K1 (Sigma, HPA039380, 1:100) primary antibody at 4 °C overnight. Slides were incubated with the secondary antibody (Alexa fluor 488 conjugated goat anti-rabbit, Invitrogen) for 1 hr. Slides were imaged with a Zeiss Axio Imager D2 microscope equipped with a high-resolution camera and Zeiss Zen23pro software.

### Assessment of visual function

Visual function in wild-type *Ppip5k2*^+*/*+^ (n = 7), *Ppip5k2*^+*/K^*^ (n = 5), and *Ppip5k2*^*K^/K^*^ (n = 3) mice was determined using the OptoMotry system (CerebralMechanics, Inc.)^87,88^. Visual acuity and contrast sensitivity are measured via rapid quantification of opto-kinetic tracking (OKT) thresholds as previously described^79^. Visual acuity was determined as the highest spatial frequency for which mice tracked a 100% contrast sine wave grating. Contrast sensitivity was identified as 100 divided by the minimum percent contrast that generated tracking at a spatial frequency of 0.064 cycles/degree. This value for spatial frequency is known to produce a robust tracking response and substantial contrast sensitivity^88^. All measurements were taken at a constant grating speed of 12 degrees/second. Measurements were performed by an investigator (BAM) blinded to mouse genotypes.

### Statistical analysis

Statistical significance was calculated using the two-tailed Student’s t-test with GraphPad Prism 8.02. A p value ≤ 0.05 was considered statistically significant.

## Supplementary information

Supplemental Data

## Data Availability

The WES and WGS datasets supporting the conclusions of this article, due to the limits stipulated in the original informed consent forms, could not be deposited into the dbGAP (the database of genotypes and phenotypes) or other public databases. The de-identified WES and WGS sequencing data may be available to qualified investigators upon request made to the Research Institutional Review Board at Augusta University by submitting to irb@augusta.edu. The gene-trap mice were purchased from the Jackson Laboratory and were available at the KOMP (the Knockout Mouse Project) Repository at the University of California, Davis. These mice are also available upon request made to the corresponding author.
