# Supplementary material for: PPIP5K2 and PCSK1 are Candidate Genetic Contributors to Familial Keratoconus
Source: Sci Rep. 2019 Dec 18;9:19406. doi: 10.1038/s41598-019-55866-5 (PMC6920454; doi:10.1038/s41598-019-55866-5)
Supplement: Supplementary file 1 — Supplemental Data [file 41598_2019_55866_MOESM1_ESM.pdf]

PPIP5K2 and _PCSK1_ are Candidate Genetic Contributors to Familial Keratoconus

Mariam Lofty Khaled[1,2#], Yelena Bykhovskaya[3#], Chunfang Gu[4], Alice Liu[5], Michelle D. Drewry[1], Zhong Chen[1], Barbara A. Mysona[1,6], Emily Parker[1], Ryan P. McNabb[5], Hongfang Yu[1], Xiaowen Lu[1], Jing Wang[1], Xiaohui Li[7], Abdulrahman Al-Muammar[8], Jerome I. Rotter[7], Louise F. Porter[9], Amy Estes[6, 10], Mitchell A. Watsky[1,6], Sylvia B. Smith[1,6,10], Hongyan Xu[11], Khaled K. Abu-Amero[8], Anthony Kuo[5], Stephen B. Shears[4], Yaron S. Rabinowitz[3$], Yutao Liu[1,6,12$]

[1] Department of Cellular Biology and Anatomy, Augusta University, Augusta, GA, USA

[2] Department of Biochemistry, Faculty of Pharmacy, Cairo University, Egypt

[3] Department of Surgery and Regenerative Medicine Institute, Cedars-Sinai Medical Center, Los Angeles, CA, USA

[4] Inositol Signaling Group, Signal Transduction Laboratory, National Institute of Environmental Health Sciences, Research Triangle Park, NC, USA

[5] Department of Ophthalmology, Duke University Medical Center, Durham, NC, USA

[6] James and Jean Culver Vision Discovery Institute, Augusta University, Augusta, GA, USA

[7] Institute for Translational Genomics and Population Sciences, Los Angeles Biomedical Research Institute and Department of Pediatrics and Medicine at Harbor-UCLA, Torrance, CA, USA

[8] Department of Ophthalmology, Glaucoma Research Chair, King Saud University, Riyadh, Saudi Arabia

[9] Department of Eye and Vision Science, University of Liverpool, and St Paul's Eye Unit, Royal Liverpool Hospital, Liverpool, UK

[10] Department of Ophthalmology, Augusta University, Augusta, GA, USA

[11] Department of Population Health Science, Augusta University, Augusta, GA, USA

[12] Center for Biotechnology and Genomic Medicine, Augusta University, Augusta, GA, USA

The first two authors marked with # are considered to have contributed to this study equally.

The final two authors marked with $ are considered to have supervised this study equally.

Corresponding author:

Yaron S. Rabinowitz

Cornea Genetic Eye Institute

50 N. La Cienega Boulevard Suite #340

Beverly Hills, CA 90211, USA

Email: yaron.rabinowitz@cshs.org

or

Yutao Liu

1460 Laney Walker Blvd CB1101

Augusta, GA 30912, USA

Phone: 1-706-721-2015

Fax: 1-706-721-2015

Email: yutliu@augusta.edu

Supplemental Figure 1. Alignment of the amino acid Serine 419 of human PPIP5K2 protein across 100 vertebrates. The amino acid S419 was highlighted in yellow. The figure indicates the relatively high conservation across all vertebrates.

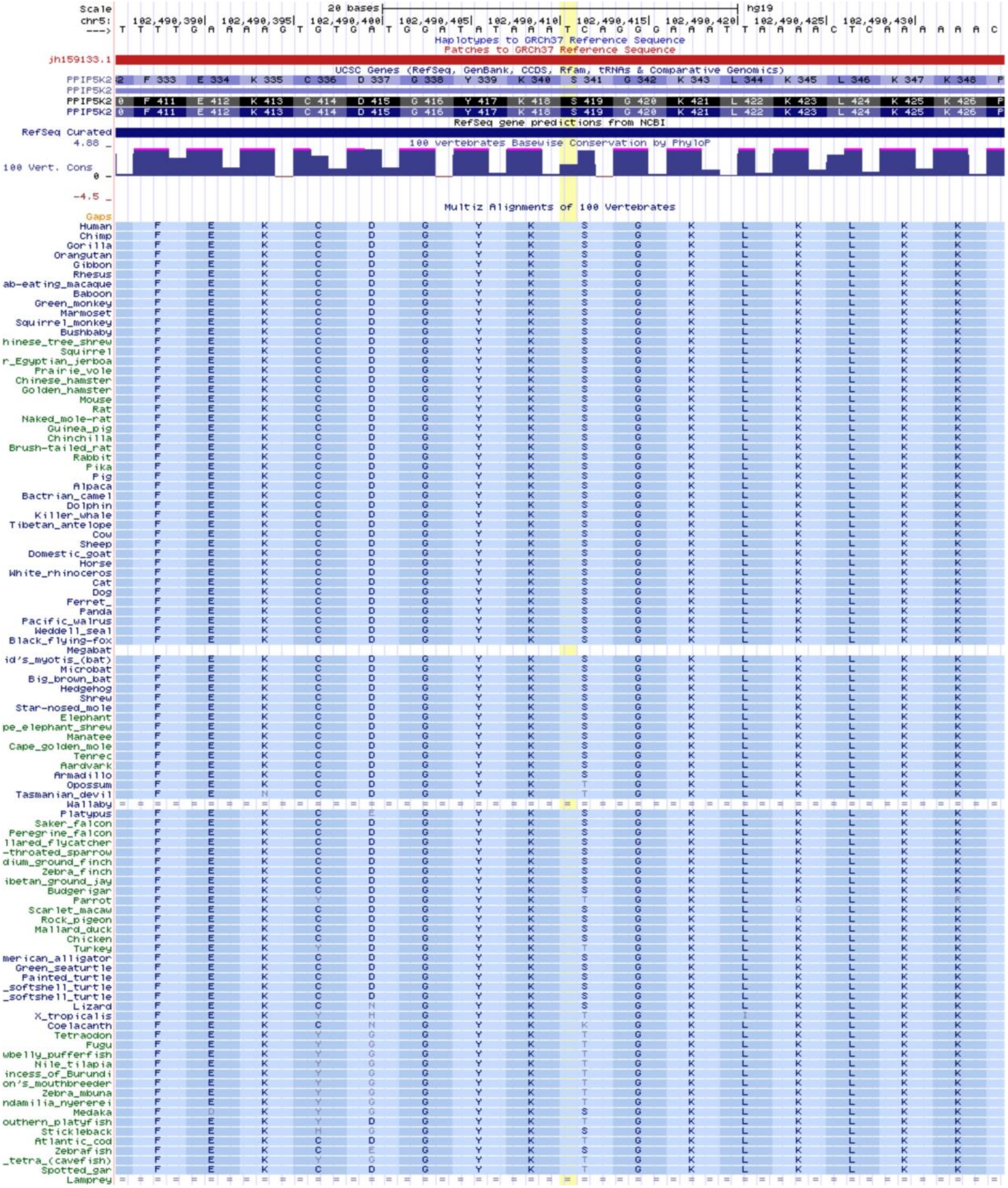

Supplemental Figure 2. Alignment of the nucleotide within the *PCSK1* gene (rs373951075, c.1096-10G>A) across 100 vertebrates. This specific nucleotide was highlighted in yellow. This figure indicates that this nucleotide is not well conserved across all the vertebrates.

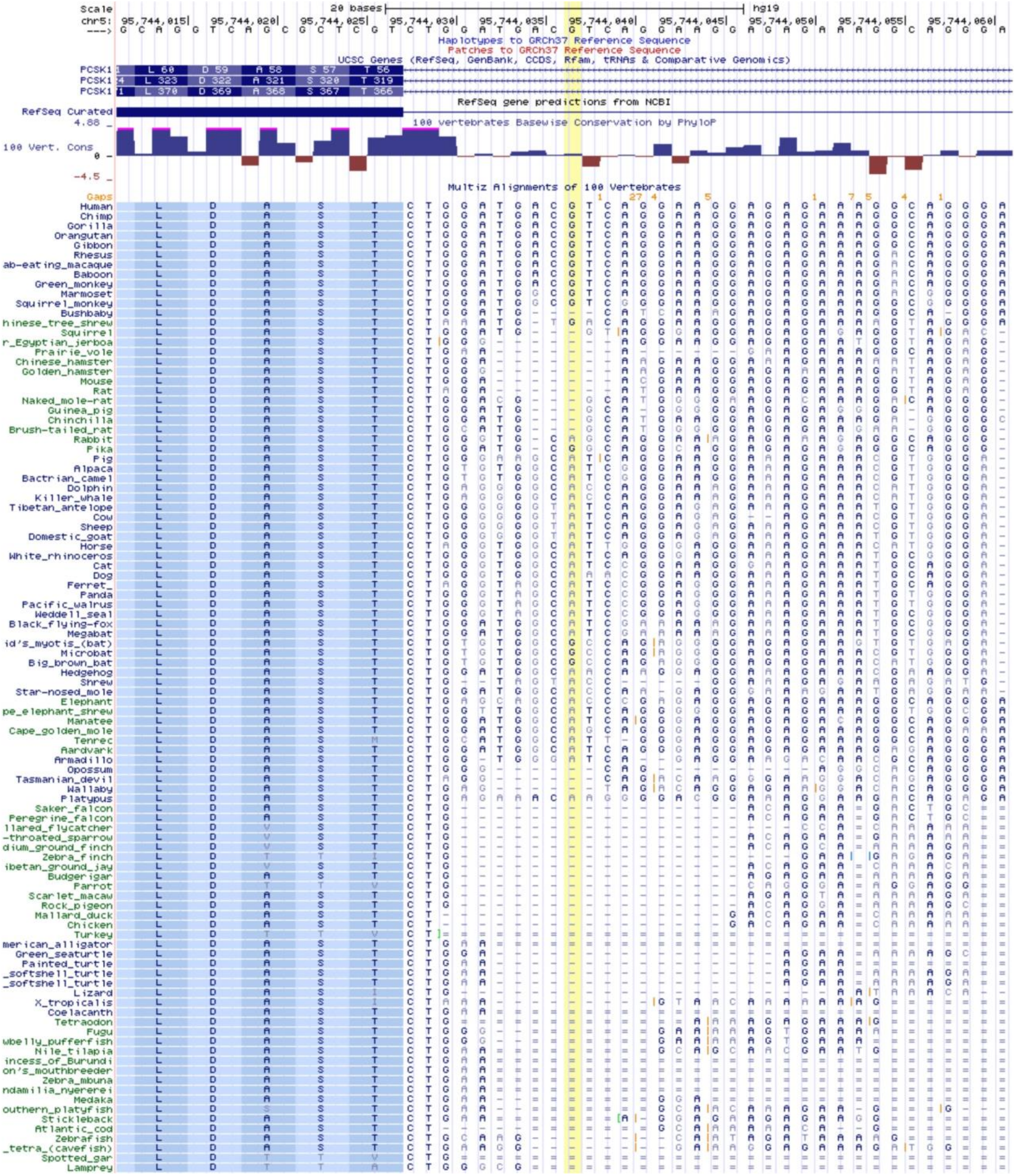

Supplemental Figure 3. Alignment of the amino acid N843 of human PPIP5K2 protein across 100 vertebrates. The amino acid N843 was highlighted in yellow. This amino acid is relatively highly conserved across all the vertebrates with either N or D amino acid.

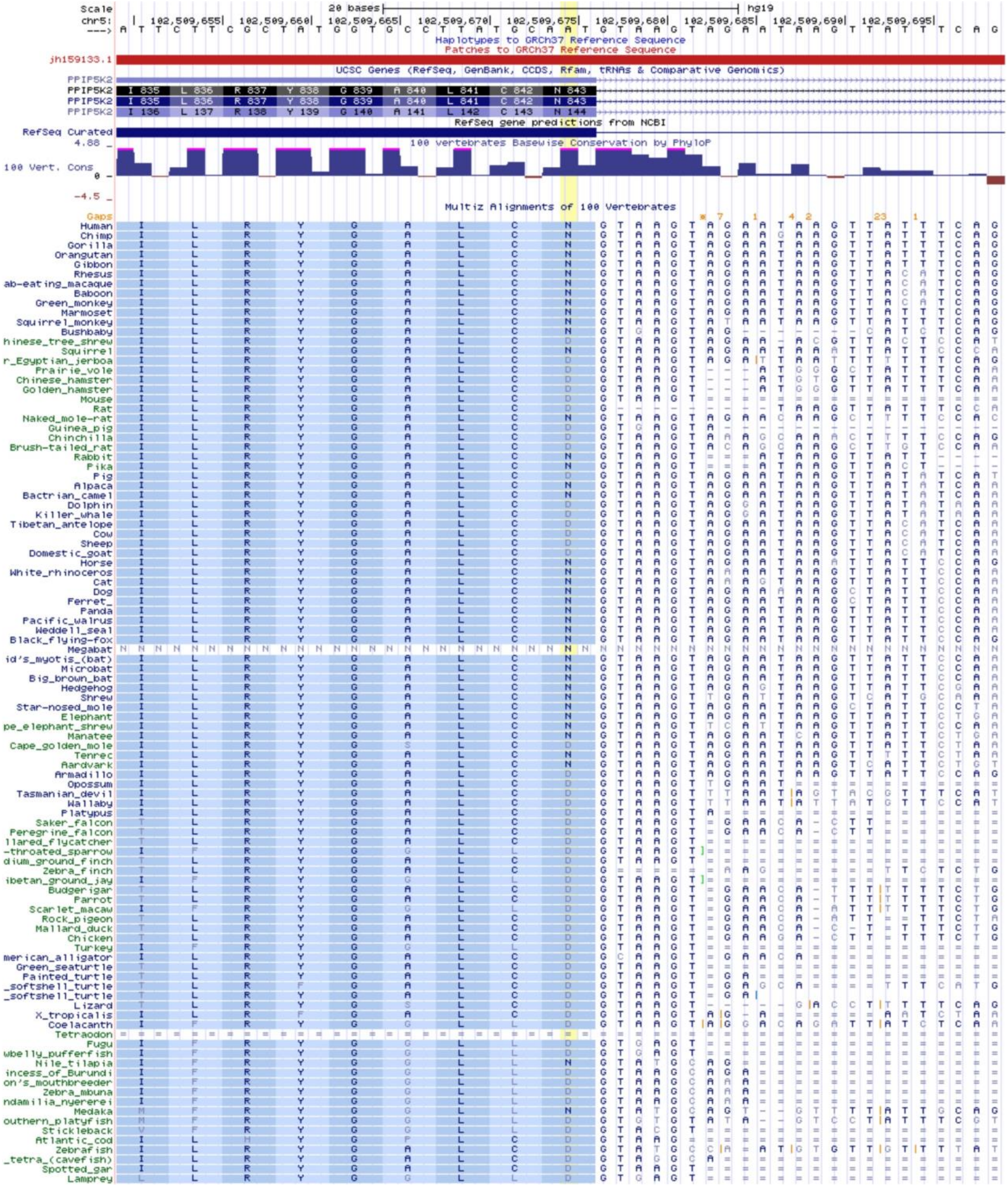

Supplemental Figure 4. Bioinformatics pipeline to filter variants derived from whole exome sequencing with 10 family members.

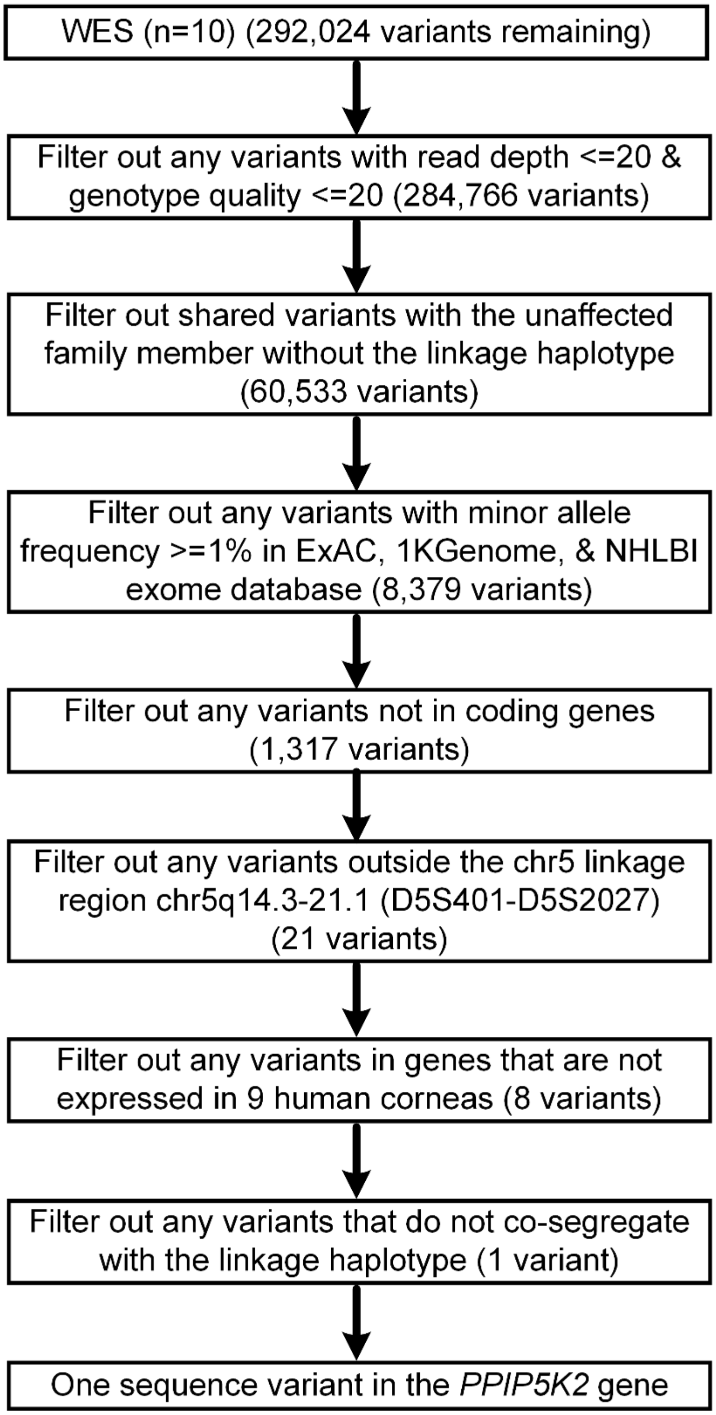

Supplemental Figure 5. Variant filtering pipeline designed for analyzing the whole genome sequencing data.

	**CA1** (KC)	**CA2** (KC)	**CA3** (KC)	**CA5** (**KC**)	**CA4** (unaffected)
# Variants after WGS	4,443,805	4,440,245	4,459,147	4,426,786	4,450,888
# Variants on Chr5	272,142	269,330	268,972	262,322	266,347
High quality and Read Depth	229,220	238,471	229,910	233,029	213,344
# Variants shared between CA1 & CA2	174,609				
# Variants shared between CA1 & CA2 & CA3	121,345				
# Variants shared between CA1 & CA2 & CA3 & CA5	99,161				
# Variants with MAF < 1%	11,993				
# Variants in coding region	2,127				
# Variants not shared with CA4 (control)	679				
# Variants in the linkage region	135				
# Variants co-segregating with the linkage haplotype	1 *PCSK1* intronic variant in the main branch of this family				
	PPIP5K2 missense variant was immediately outside the linkage region				

Supplemental Table 1. List of individuals with their clinical phenotypes and genotypes in the four-generation keratoconus family

ID	Family ID	Individual ID	Father ID	Mother ID	Sex	Affectious status	Grey haplotype	Genotype for PPIP5K2 c.1255	Genotype for PCSK1 c.1096-10
KC1	II-4	206	101	102	2	2	YES	T/G	G/A
KC2	II-5	202	101	102	1	2	YES	T/G	G/A
KC3	II-6	216	0	0	2	1	NO	T/T	G/G
KC4	III-2	302	201	202	2	2	NO	T/T	G/G
KC5	III-5	311	205	206	1	2	YES	T/G	G/A
KC6	III-6	318	0	0	1	1	NO	T/T	G/G
KC7	III-7	301	205	206	2	2	YES	T/G	G/A
KC8	III-8	321	0	0	1	1	NO	T/T	G/G
KC9	III-9	310	205	206	2	2	YES	T/T	G/A
KC10	III-10	303	202	216	1	1	YES	T/G	G/A
KC11	III-11	304	0	0	2	1	NO	T/T	G/G
KC12	III-12	305	202	216	2	1	YES	T/G	G/A
KC13	III-13	343	0	0	1	1	NO	T/T	G/G
KC14	III-14	317	202	216	1	2	YES	T/G	G/A
KC15	IV-1	403	303	302	1	1	NO	T/T	G/G
KC16	IV-2	401-400	304	301	1	2	NO	T/T	G/G
KC17	IV-3	418	311	320	1	1	YES	T/G	G/A
KC18	IV-4	423	311	320	2	2	NO	T/T	G/G
KC19	IV-5	422	311	320	1	2	YES	T/G	G/A
KC20	IV-6	440	311	320	1	2	NO	T/T	G/G
KC21	IV-7	409	318	301	2	1	YES	T/G	G/A
KC22	IV-8	401	321	310	2	1	NO	T/T	G/G
KC23	IV-9	425	321	310	2	1	YES	T/T	G/A
KC24	IV-10	404	303	304	1	2	YES	T/G	G/A
KC25	IV-11	408	303	304	2	1	NO	T/T	G/G
KC26	IV-12	419	343	305	1	2	YES	T/G	G/A

Note: Sex 1 for male and 2 for female; Affection status 1 for unaffected and 2 for affected with keratoconus; KC for keratoconus.

Supplemental Table 2. List of individuals with their clinical phenotype and genotypes in the second keratoconus family

ID	Family ID	Individual ID	Father ID	Mother ID	Sex	Affection status	PPIP5K2 c.2528A>G
US-KC1	914	201	unknown	unknown	2	1	A/G
US-KC2	914	202	unknown	unknown	1	1	A/G
US-KC3	914	301	202	201	1	2	A/G
US-KC4	914	302	202	201	2	1	A/G
US-KC5	914	303	202	201	2	2	A/G
US-KC6	914	304	202	201	1	1	A/G
US-KC7	914	305	202	201	1	1	A/G
US-KC8	914	306	202	201	2	1	A/A
US-KC9	914	307	202	201	2	1	A/A
US-KC10	914	308	202	201	2	1	A/A

Note: Sex 1 for male and 2 for female; Affection status 1 for unaffected and 2 for affected with keratoconus; KC for keratoconus.
